# Supplementary figures and images for: Nest site selection and nutritional provision through excreta: a form of parental care in a tropical endogeic earthworm
Source: PeerJ. 2016 May 17;4:e2032. doi: 10.7717/peerj.2032 (PMC4878366; doi:10.7717/peerj.2032)

1  
2  
3  
4  
5  
6  
7

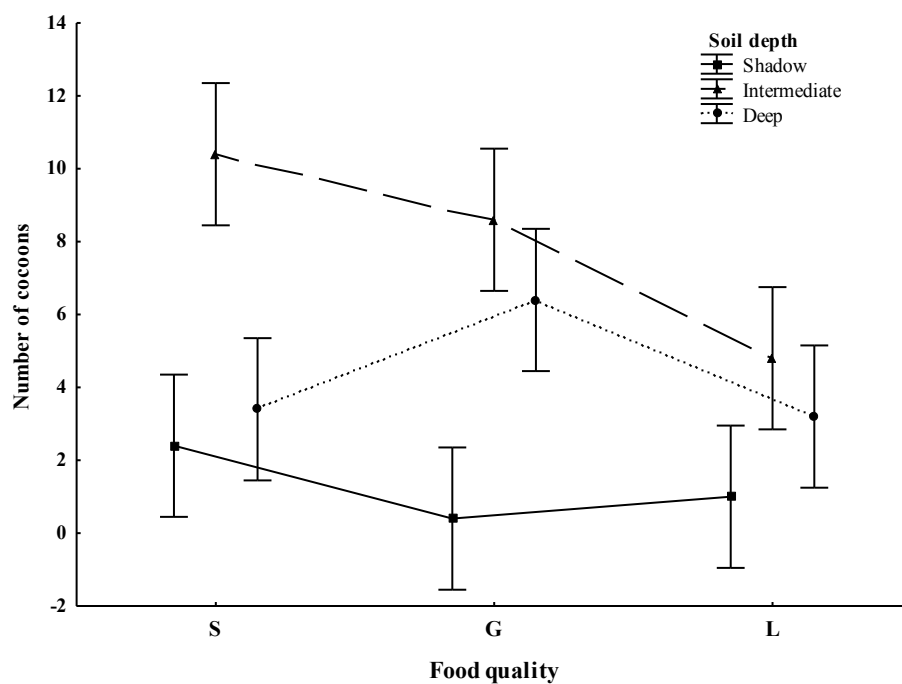

8

Supplement: Figure S1 — Soil depth: Shadow = 0–9 cm, Intermediate = 10–18 cm, Deep = 19–27 cm. Soil Quality: S = soil only, G = soil + grass, L = soil + legume. Vertical lines indicate 95% confidence intervals. [file peerj-04-2032-s002.pdf]

1

2

3

4

5

6

7

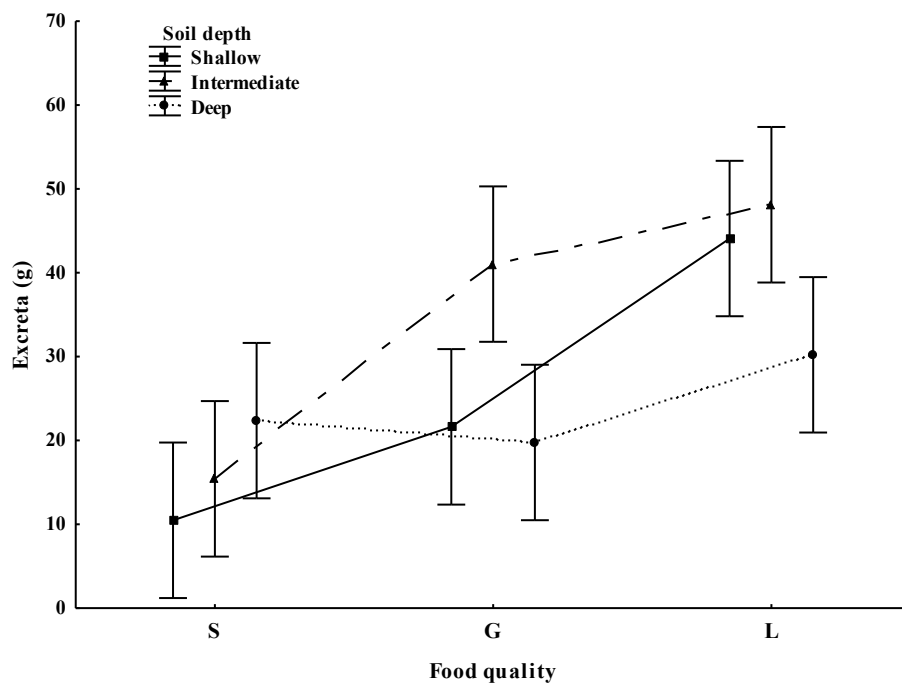

8

Supplement: Figure S2 — Soil depth: Shadow = 0–9 cm, Intermediate = 10–18 cm, Deep = 19–27 cm. Soil Quality: S = soil only, G = soil + grass, L = soil + legume. Vertical lines indicate 95% confidence intervals. [file peerj-04-2032-s003.pdf]

1  
2  
3  
4  
5

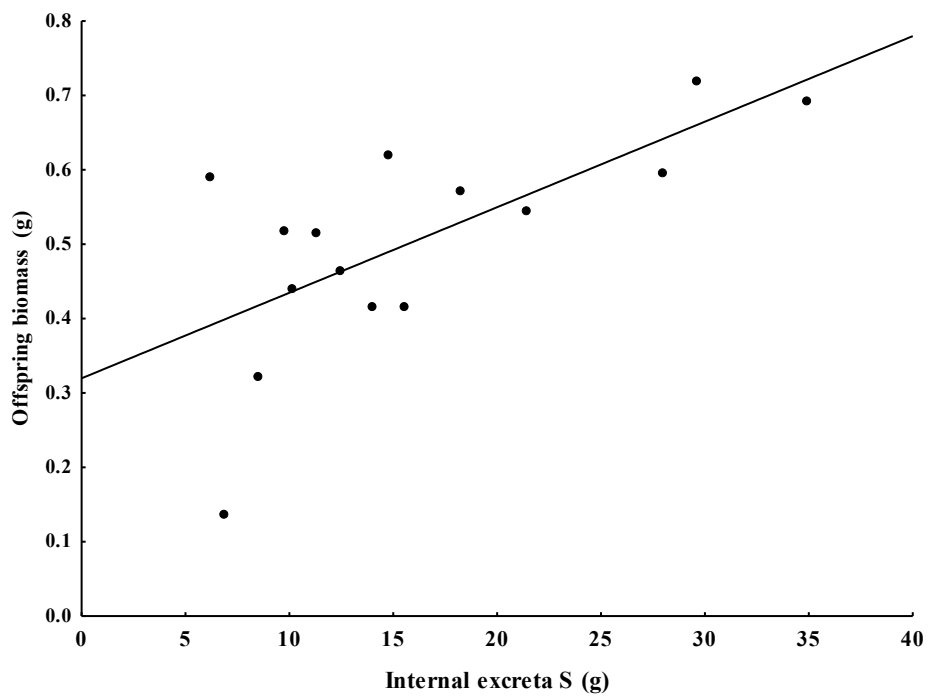

6  
7  
8

Supplement: Figure S3 — S = only Soil. The line in fitted with a linear regression. [file peerj-04-2032-s004.pdf]
